# The Polyphenols α-Mangostin and Nordihydroguaiaretic Acid Induce Oxidative Stress, Cell Cycle Arrest, and Apoptosis in a Cellular Model of Medulloblastoma

**DOI:** 10.3390/molecules26237230

**Published:** 2021-11-29

**Authors:** Alberto Rojas-Ochoa, Emilio J. Córdova, Adela Carrillo-García, Marcela Lizano, José Pedraza-Chaverri, Nelly Patiño, Alfredo Cruz-Gregorio, Norma Osnaya

**Affiliations:** 1 Laboratorio de Oncología Experimental, Subdirección de Medicina Experimental, Instituto Nacional de Pediatría, Ciudad de México 04530, Mexico; 2 Consorcio de Oncogenómica, Instituto Nacional de Medicina Genómica, Ciudad de México 14610, Mexico; ecordova@inmegen.gob.mx; 3 Subdirección de Investigación Básica, Instituto Nacional de Cancerología, Ciudad de México 14080, Mexico; acarrillog@incan.edu.mx (A.C.-G.); lizano@unam.mx (M.L.); 4 Departamento de Medicina Genómica y Toxicología Ambiental, Instituto de Investigaciones Biomédicas, Universidad Nacional Autónoma de México, Ciudad de México 04510, Mexico; 5 Departamento de Biología, Facultad de Química, Universidad Nacional Autónoma de México, Ciudad de México 04510, Mexico; pedraza@unam.mx (J.P.-C.); cruzgalfredo@comunidad.unam.mx (A.C.-G.); 6 Unidad de Citometría de Flujo (UCiF), Instituto Nacional de Medicina Genómica, Ciudad de México 14610, Mexico; lnpatino@inmegen.gob.mx; 7 Laboratorio de Neurociencias, Subdirección de Medicina Experimental, Instituto Nacional de Pediatría, Ciudad de México 04530, Mexico; nosnayab@pediatria.gob.mx

**Keywords:** medulloblastoma, α-mangostin, NDGA, cancer, cell cycle, apoptosis

## Abstract

Medulloblastoma is a common malignant brain tumor in the pediatric age. The current therapeutics present serious collateral effects. Polyphenols α-mangostin and nordihydroguaiaretic acid (NDGA) exert potent antitumoral activity in different cancer models, although their antitumoral effects have not been described in medulloblastoma cells yet. This study aimed to examine the proapoptotic effects of these polyphenols on human medulloblastoma cells. Medulloblastoma cell line Daoy was incubated with increasing concentrations of α-mangostin or NDGA for 24 h. The cell viability was analyzed using crystal violet and trypan blue dyes. Determination of the glutathione (GSH)/glutathione disulfide (GSSG) ratio and levels of carbonylated proteins was performed to evaluate the oxidative stress. Cell cycle progression and induction of cell death by fluorochrome-couple and TUNEL assays were evaluated using flow cytometry assays. Individual treatments with α-mangostin or NDGA decreased the viability of Daoy cells in a dose-dependent manner, inducing G2/M and S-G2/M cell cycle arrest, respectively. Both polyphenols induced cell death and increased oxidative stress. Very interestingly, α-mangostin showed more potent effects than NDGA. Our results indicate that α-mangostin and NDGA exert important cytostatic and cytotoxic effects in the Daoy cell line. These data highlight the potential usefulness of these compounds as an alternative strategy in medulloblastoma treatment.

## 1. Introduction

Medulloblastoma, a malignant tumor that develops in the cerebellum, is considered the most frequent cerebral cancer in the pediatric age, accounting for approximately 20% of all primary neoplasia of the central nervous system [1]. This tumor is diagnosed in children younger than 15 years old, usually between 3 and 9. The current therapeutic schemes used in medulloblastoma patients are based on neurosurgery, craniospinal radiotherapy, and conventional chemotherapy [2]. Although recent therapeutic advances have increased the survival rate of patients, a significant number of collateral adverse effects related to these procedures are detrimental to the quality of life of the survivors [2,3]. These side effects include important affectation and deterioration of intellectual function and memory, motor ability deficits, and growth problems [1,2,3]. Therefore, the discovery of nontoxic therapeutic alternatives against medulloblastoma is still an area of active research.

For centuries, plant-derived compounds have been used in traditional medicine to cure a diversity of human diseases [4,5]. In this regard, mainly two plants have great potential as therapeutic alternatives, the tropical tree native to Southeast Asia known as mangosteen (*Garcinia mangostana, Linn*) and a creosote bush known as La Gobernadora (*Larrea tridentata*) or Hediondilla in México and greasewood or chaparral in the United States of America, because both plants have a long history of use in traditional medicine in their original regions. These plants are rich in polyphenols with potent chemopreventive properties and very few or no adverse side effects linked to their consumption in treating various health conditions like inflammation, neurological disorders, aging, arthritis, diabetes, obesity, renal disease, and cancer [6,7,8,9,10,11]. The abundant polyphenols α-mangostin from the fruits produced by mangosteen and nordihydroguaiaretic acid (NDGA) from La Gobernadora have been studied, because they possess excellent beneficial health properties, such as ameliorative effects in cardiovascular and neurological disorders, immune modulation, antiviral effect, tissue engineering, and solid anticancer function [12,13,14,15]. The majority of in vivo studies of mangosteen xanthones have focused on murine and rat models and included metabolism and bioavailability analyses, particularly in anticancer activity, which have focused on colon cancer [16]. Remarkably, α-mangostin has been shown to inhibit cell proliferation in prostate and breast cancer-derived cell lines [17,18].

Moreover, the proapoptotic properties of α-mangostin have been demonstrated in many cancer cellular models. For instance, in prostate cancer cells, α-mangostin modulates the endoplasmic reticulum stress (ER) markers, protein kinase RNA-like endoplasmic reticulum kinase (PERK), inositol requiring enzyme (IRE1), and C/EBP homologous protein (CHOP) with the activation of caspase-3, whereas, in breast cancer cells, it activates caspase-8, caspase-9, and caspase-7. In colorectal cancer cells, α-mangostin increases the protein levels of the proapoptotic molecules tumor suppressor protein p53, the pro-apoptotic BCL2 gene family member BAX (BAX), poly (ADP-ribose) polymerase (PARP), and the release of Endonuclease-G (Endo-G) from the mitochondria. Moreover, in leukemia-derived cell lines, α-mangostin induces nucleosomal DNA fragmentation and the activation of caspase-9 and caspase-3 [17,18,19,20,21]. Besides, α-mangostin exposure reduces the migration and invasion potential of cancer cells derived from pancreatic carcinoma (BxPc-3 and Panc-1) and melanoma (B16-F10) [22,23]. On the other hand, it has been shown that α-mangostin at 100–1000 mg/kg did not exhibit any harmful effects on the kidney and liver tissues of murine models [24] and did not affect the cell viability in normal human osteoblasts when treated by α-mangostin (0, 10, 20, 30, 40, and 50 μM) for 24 and 48 h [25].

NDGA cytotoxic and antitumoral activities have been evaluated, both in cell cultures and in animal models. For instance, NDGA promotes cell death by caspase-3 cleavage in the breast cancer cell lines SK-BR-3 and BT-474 [26]. In addition, NDGA inhibits growth and induces cell cycle arrest at the G1 phase in cervical cancer-derived cell line SiHa by regulating cell cycle kinase inhibitor p21 and promoting the acetylation of histone H3 [27]. In the prostate cancer PC3 cell line, NDGA impairs cell migration and tumor metastasis in nude mice via suppressing the function of single-pass transmembrane protein neuropilin 1 [28]. Regarding the toxic effect of NDGA in no cancer cells, it has been reported that only high concentrations of NDGA (100 μM) induce lipid peroxidation, DNA double-stranded breaks, and cell death in rat hepatocytes [29] and do not affect the viability of normal cells and peripheral blood mononuclear cells (PBMC) at 3, 10, 30, and 60 μM [30].

There have been several reports of studies with healthy human volunteers where extracts, juices, or teas are used, either from the fruits of mangosteen or of La Gobernadora leaves; in these reports, the bioavailability and metabolism of the compounds containing these plants were studied primarily [9,16,31,32,33]. However, although there are as yet no reports of studies related to their neuroprotective and anticancer activities in humans with mangosteen or α-mangostin, there have been some clinical trials with NDGA or its derivates for various cancer types like prostate, cervical, leukemia, and glioma, though no conclusive results have been reported [34]. For instance, in a terminated phase II clinical trial, subjects with nonmetastatic hormone-sensitive prostate cancer (HSPC) were treated with oral NDGA; the patients presented no significant prostate-specific antigen (PSA) declines after three cycles of treatment, so the researchers did not recommend NDGA as a monotherapy in this population [35]. In another completed phase I clinical trial, the intravenously NDGA analog called Terameprocol defined the toxicity profile in men with recurrent high-grade gliomas; this phase I study defined the dose for future studies with terameprocol and suggested that safely combining this compound with chemotherapy drugs and with radiation to study its synergistic effects in newly diagnosed high-grade gliomas could be accomplished [36].

Although α-mangostin and NDGA have been proposed as potential alternatives in treating different types of cancer, there have been no reports of the anticancerous effects of these polyphenols during human in vivo models or even in vitro models of medulloblastoma. Thus, the present study aimed to evaluate the cytostatic and cytotoxic effects of α-mangostin and NDGA in the childhood medulloblastoma-derived cell line Daoy, which belongs to Sonic Hedgehog (SHH), which is the second-most common subgroup in children under three years old and adolescents in approximately 33% of all the subgroups. Our results showed that both α-mangostin and NDGA exerted cytostatic and cytotoxic effects in the Daoy cell line, probably by the oxidative stress produced for these polyphenols, with α-mangostin having more substantial effects than NDGA. These polyphenols provoked arrest in different stages of the cell cycle, suggesting the involvement of independent cellular mechanisms, and we also observed that, importantly, these compounds induced apoptosis in medulloblastoma cell line Daoy. Our data highlighted the potential usefulness of these compounds as an alternative strategy in medulloblastoma treatment.

## 2. Results

### 2.1. Effects of α-Mangostin and NDGA on Cell Viability in Daoy Cells

**Figure 1:**
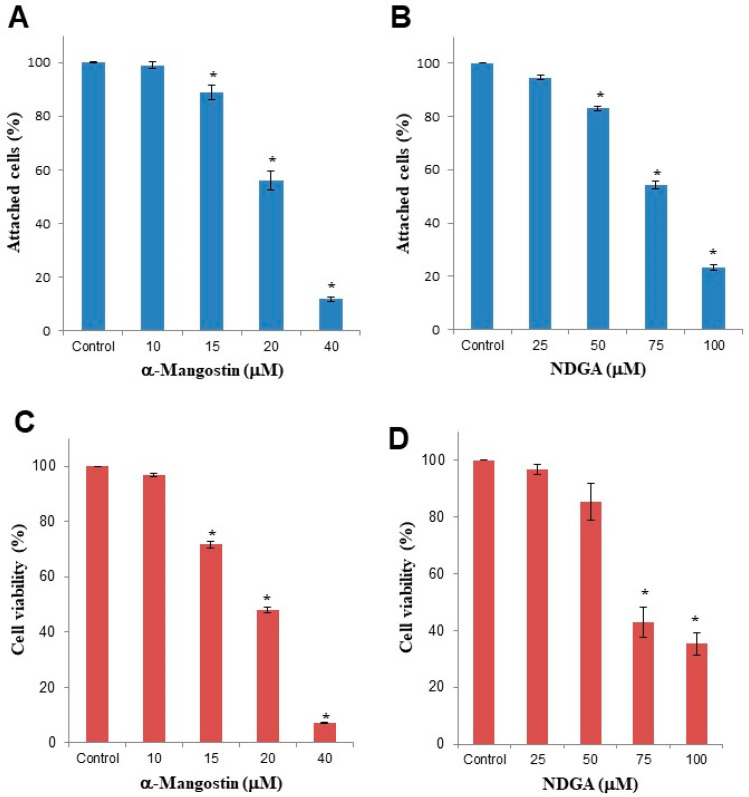
Cellular growth and viability of Daoy cells were decreased by individual treatments with α-mangostin or NDGA. The Daoy cell line (1 × 10^5^ cells/mL) was cultured for 24 h in the presence of increasing concentrations of α-mangostin or NDGA. (**A**,**B**) The percentage of adherent cells was assessed with a crystal violet assay. (**C**,**D**) Cellular viability was determined by the trypan blue exclusion assay. Data represent the mean and standard error (SEM) of at least three independent experiments. One-way analysis of variance and Dunnett’s multiple comparison test were used. * *p* < 0.0001 vs. control.

We first determined the effect of increasing concentrations of α-mangostin and NDGA on the viability of medulloblastoma cells. We found a dose-dependent reduction in the number of attached cells in α-mangostin and NDGA-treated cell cultures (Figure 1A,B). However, this decrease was observed with lower concentrations of α-mangostin (15 μM = 88.93%, 20 μM = 55.92%, and 40 μM = 11.88%) than NDGA (50 μM = 83.09%, 75 μM = 54.19%, and 100 μM = 23.19%).

Using the trypan blue exclusion assay, we also observed a dose-dependent decrease in cellular viability after α-mangostin (15 μM = 71.71%, 20 μM = 48.15%, and 40 μM = 7.32%) or NDGA treatments (50 μM = 85.48, 75 μM = 42.78%, and 100 μM = 35.31%) (Figure 1C,D). The reduction in cell viability was observed again, with lower concentrations of α-mangostin compared to NDGA. These data indicated a more potent effect of α-mangostin than NDGA on Daoy cell growth.

### 2.2. The α-Mangostin and NDGA Promote Oxidative Stress in Daoy Cells

**Figure 2:**
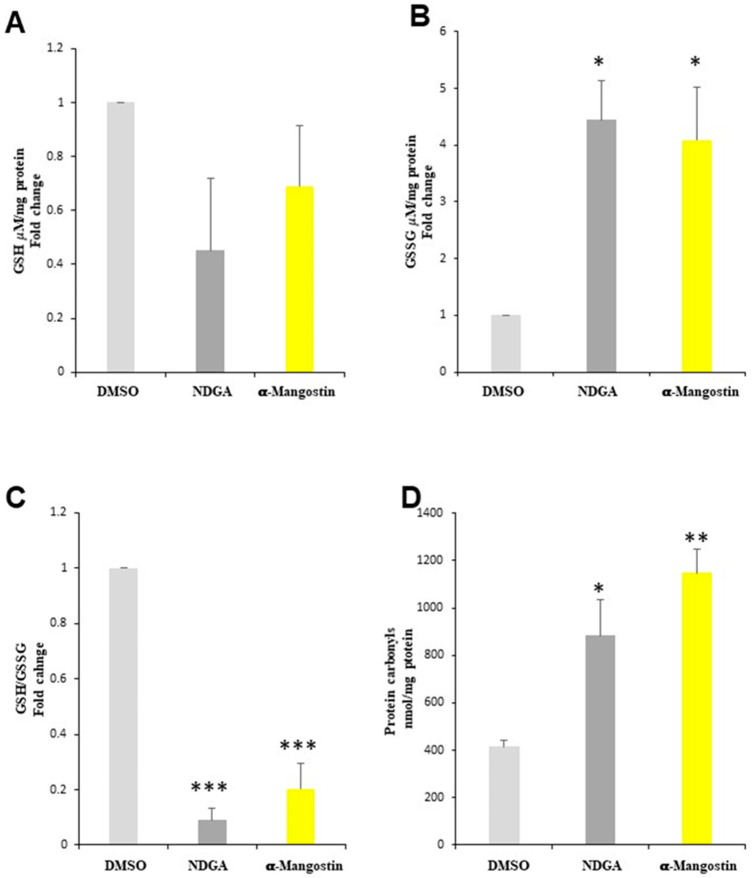
α-Mangostin (20 μM) and NDGA (75 μM) promote oxidative stress in Daoy cells. (**A**) GSH determination, (**B**) GSSG determination, (**C**) GSH/GSSG ratio compared to the control vector, and (**D**) carbonyl protein determination compared to the control vector. The GS, and GSSG levels are expressed as the mean and SEM. Tukey’s test: * *p* < 0.05 and *** *p* < 0.0005. The carbonyl protein levels are expressed as the mean and SEM. Tukey’s test: * *p* < 0.05 and ** *p* < 0.005.

After observing that α-mangostin and NDGA affected the cell number and viability in the cell cultures, and knowing that, in some conditions, these compounds can induce oxidative stress (OS), which is one of the mechanisms that induce cell death, we measured the intracellular levels of glutathione and carbonyl proteins in Daoy cells treated with α-mangostin and NDGA as markers of OS. We observed a nonsignificant decrease in the glutathione levels (Figure 2A), as well as a 4- and 4.5-fold increase of GSSG, after the α-mangostin and NDGA treatments, respectively (Figure 2B). Consequently, the GSH/GSSG ratio decreased 4.9- and 11-fold for α-mangostin and NDGA, respectively (Figure 2C). Additionally, we found that the level of carbonyl protein groups increased with α-mangostin and NDGA treatments 2.8- and 2.1-fold, respectively (Figure 2D). Taken together, these results suggest that α-mangostin and NDGA treatments induce oxidative damage to proteins, which might be a possible mechanism of cell death induction by α-mangostin and NDGA in the Daoy cell line.

### 2.3. α-Mangostin and NDGA Induced Arrest in Different Stages of the Cell Cycle in Daoy Cells

**Figure 3:**
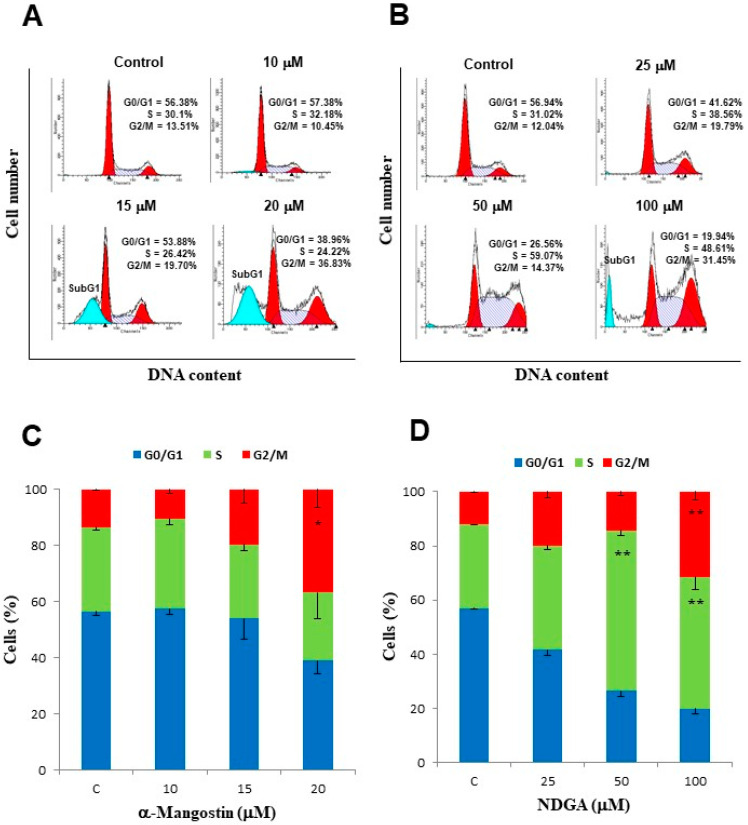
Treatment with α-mangostin and NDGA induced cell cycle arrest in Daoy cells. Daoy cells (1 × 10^6^ cells) were treated with increasing doses of α-mangostin or NDGA, stained with propidium iodide, and analyzed by flow cytometry. (**A**,**B**) Representative histograms and the mean of at least three independent assays are shown. (**C**,**D**) Graphical representation of the cell cycle data in panels A and B. Data represent the mean and SEM of at least three independent experiments. Two-way ANOVA and Bonferroni posttests: * *p* < 0.05 and ** *p* < 0.001.

Since we found that treatment with α-mangostin and NDGA induces OS in Daoy cells in a culture, we decided to analyze whether that OS had any effect on the cell cycle of Daoy cells in the culture; next, we evaluated the effect of each polyphenol on the cell cycle progression with flow cytometry and DNA staining with propidium iodide using dose–response assays. After incubation with 15 μM of α-mangostin for 24 h, we observed a slight increase in the percentage of Daoy cells at the G2/M phase in comparison with the control cell cultures (19.70% vs. 13.51%), whereas an evident cell cycle arrest in the G2/M phase was observed after incubation with 20 μM of α-mangostin for 24 h (36.83% vs. 13.51%). No differences in cell cycle progression were observed in the cultures incubated with 10 μM of α-mangostin in comparison with the control cultures (13.51% vs. 10.45%) (Figure 3A,C). It is worth noting that a subG0/G1 peak, representing the apoptotic cell population, was observed in the treatments with 15 and 20 μM of α-mangostin (22.81% and 25.31%, respectively), but it was not observed in the treatment with 10 μM of α-mangostin (Figure 3A).

In the case of NDGA, we found an initial increase in the percentage of Daoy cells at the S phase after treatment with 50 μM in comparison with the control cell cultures (59.07% vs. 31.02%), followed by a slight decrease in the percentage of cells in this phase at 100 μM (48.61%), whereas the percentage of cells in the G2/M phase showed a dose-dependent increase at 50 μM (14.37%) and 100 μM (31.45%) in comparison with the control cultures (12.04%) (Figure 3B,D). Like α-mangostin, we also observed a subG0/G1 peak, but only in the treatment with the highest concentration of NDGA (100 μM). The above data suggest that α-mangostin and NDGA affect different mechanisms in regulating the cell cycle.

### 2.4. α-Mangostin and NDGA Promoted Cell Death in Daoy Cells by Triggering Apoptosis

**Figure 4:**
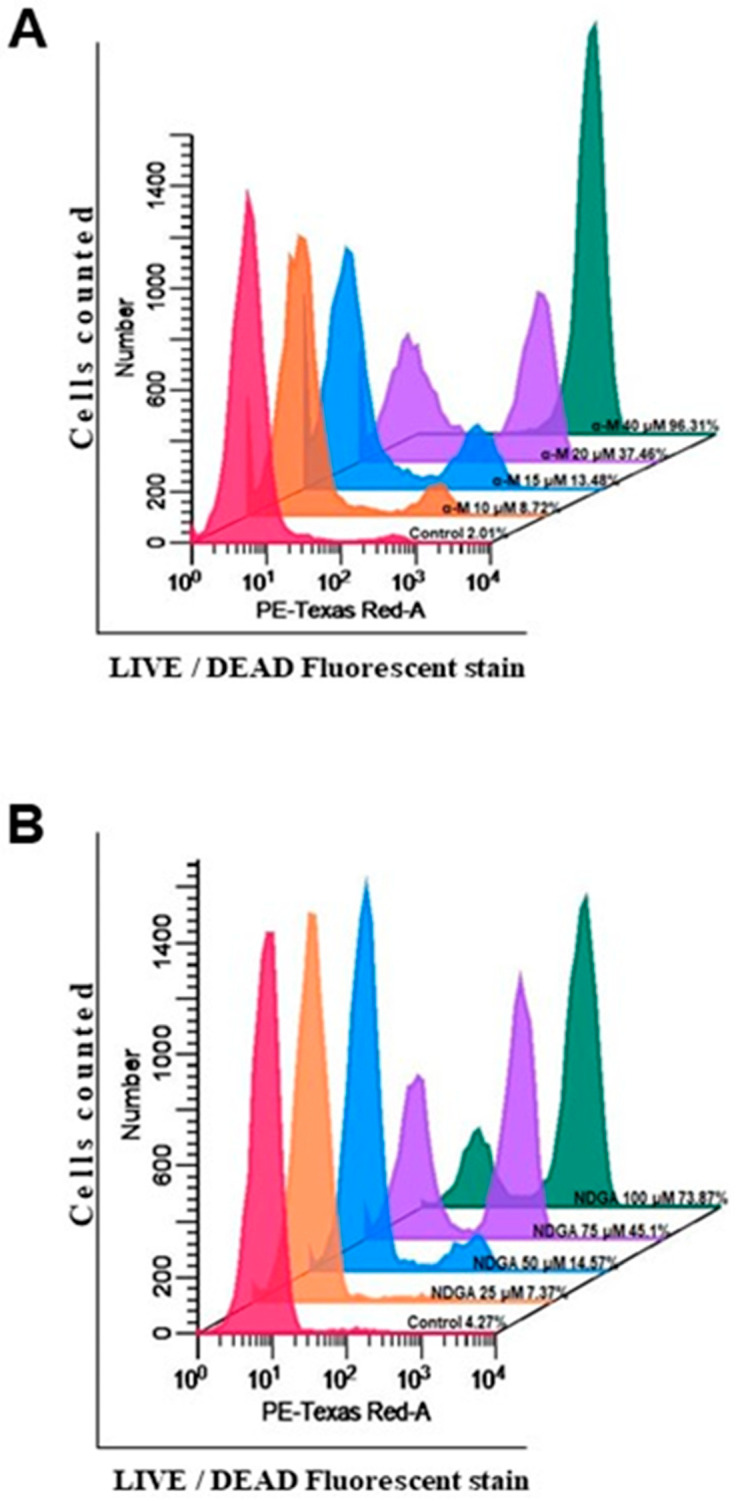
α-Mangostin and NDGA induced cytotoxicity in medulloblastoma cells. The cytotoxic effects of α-mangostin and NDGA treatment on Daoy cells were determined by LIVE/DEAD assay fluorescent staining. (**A**,**B**) Representative histograms of the cytometer determinations are shown. Mean and SEM of at least three independent experiments were analyzed with one-way analysis of variance and Dunnett’s multiple comparison test.

As the sub G0/G1 peak detected in the cell cycle assays suggested the presence of apoptosis in Daoy cells treated with these polyphenols, we used a nonpermeable fluorescent probe to detect by flow cytometry the loss of plasma membrane integrity, which is a characteristic of cell death. After 24 h of treatment with increasing concentrations of α-mangostin, we observed a concentration-dependent increase in the percentage of cells with a loss of plasma membrane integrity, starting at concentrations as low as 15 μM (20.80%) and having the highest percentage of cell death at 40 μM (99.75%) (*p* < 0.0001 vs. control) (Figure 4A). In contrast, NDGA treatment induced a significant increase in cell death only at the two highest concentrations used in this assay (75 μM = 45.10% and 100 μM = 73.87%) (*p* < 0.0001 vs. control) (Figure 4B).

**Figure 5:**
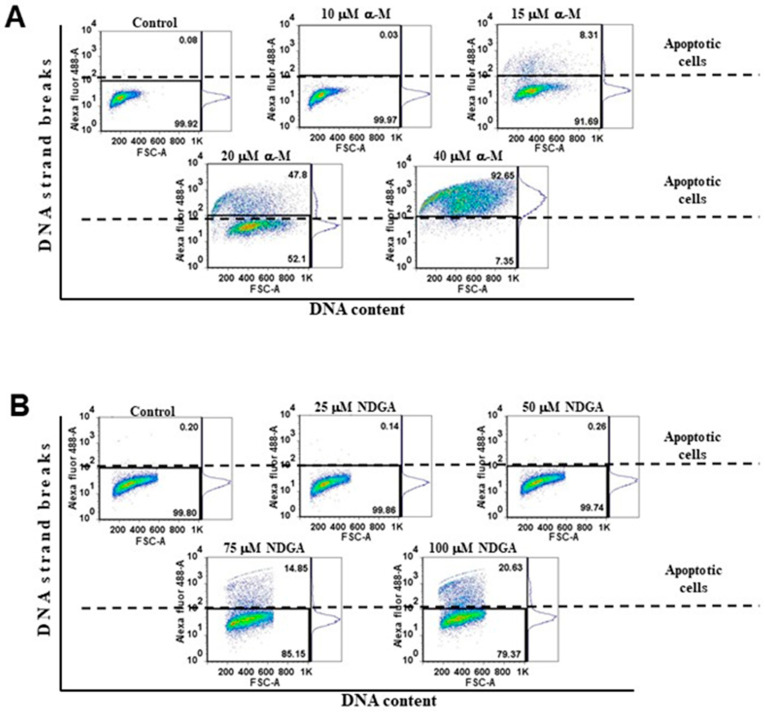
TUNEL assay of the dose–response curves of α-mangostin and NDGA on Daoy cells. (**A**,**B**) Representative histograms (dot plots) are shown. The Q1 region in the dot plots represents TUNEL-positive staining cells with the APO-BrdUTM TUNEL Assay kit. Mean and SEM of at least three independent experiments were analyzed with one-way analysis of variance and Dunnett’s multiple comparison test.

A TUNEL assay was performed to corroborate the presence of apoptosis. This assay detects the fragmentation of chromosomal DNA, one of the hallmarks of late apoptosis. As expected, we observed a significant increase in the percentage of apoptotic cells at increasing concentrations of α-mangostin and NDGA (Figure 5A,B). Following the assays related to membrane integrity, apoptotic cells began to emerge at α-mangostin concentrations of 15 μM (8.31%), showing concentration-dependent increases at 20 μM (47.81%) and 40 μM (92.65%) (*p* < 0.0001 vs. control). In contrast, the concentration of 10 μM showed no significant increase in the percentage of cell death (Figure 5A). In the case of NDGA, the concentrations of 75 μM and 100 μM induced a marked dose-dependent increase in the percentage of apoptotic cells (14.85% and 20.63%, respectively) compared to the control cultures (*p* < 0.0001 vs. control) (Figure 5B). It is worth noting that the percentage of apoptotic cells induced by 20 μM of α-mangostin was higher than that induced by 100 μM of NDGA (47.81% vs. 20.63%). Overall, our data indicated that α-mangostin has a more potent proapoptotic effect than NDGA in Daoy cells.

## 3. Discussion

Medulloblastoma is the most common cerebral malignant tumor in the pediatric age, and the current therapeutic strategies present severe side effects. Recently, several studies with dietary polyphenols such as α-mangostin and NDGA have discovered their potential functions in preventing and treating many types of cancer [12,13,14,15]. However, no studies about the antitumoral effects of these polyphenols in childhood medulloblastoma cells have been performed yet. Here, we report, for the first time, the cytostatic and pro-apoptotic effects of α-mangostin and NDGA in Daoy cells, a medulloblastoma-derived cell line.

We found an inhibitory effect of α-mangostin on cell growth in the Daoy cell line with a 50% reduction after 24 h of exposure to 20 μM of the phytochemical and complete suppression of cell growth after incubation with 40 μM. According to our findings, previous studies have reported the ability of α-mangostin to decrease cell growth near to 50% in human cancer cells, which ranged from 3.57 μM in breast cancer to 20 μM in colon carcinoma [37,38,39,40]. In the case of NDGA, the inhibitory effect on cell growth was at higher concentrations, ranging from 50 to 200 μM, where 50% inhibitory cell growth was found near 75 μM. The inhibitory effect of NDGA in cell growth has been reported in cervical and breast cancer cells in a concentration-dependent manner from 20 to 100 μM [26,27].

We also found that α-mangostin and NDGA induce a significant decrease in the GSH/GSSG ratio associated with the augmentation of carbonyl proteins in Daoy cells. This condition involves the increase of reactive oxygen species (ROS). Our results matched the effect of α-mangostin in cancer cell lines, as shown by Lee et al. [41]. This group showed that α-mangostin increased the ROS in cancer cell lines, resulting in loss of the mitochondrial membrane potential (MMP), in the increase of proapoptotic protein Bax, and the decrease of antiapoptotic proteins such as Bcl-2, which induce cytochrome C release and activation of caspase-9/caspase-3, promoting apoptosis [41].

In addition, after exposing the Daoy cell line to 20 μM of α-mangostin for 24 h, we observed G2/M cell cycle arrest. In contrast, most of the studies reporting the cytostatic effects of α-mangostin in cellular cancer models showed an arrest at the G1 phase, using concentrations ranging from 8 μM to 20 μM in different incubation periods. For instance, in pancreatic cancer cells BxPc-3 and Panc-1, α-mangostin treatment induced a decrease of cyclin-D1 followed by cell cycle arrest at the G1 phase [22]. Similarly, in B16-F10 melanoma cells, 15 μM of α-mangostin induced G1 arrest after 24 h of treatment [23]. Moreover, in cell lines derived from oral squamous cell carcinoma (HSC-2, HSC-3, and HSC-4) and colon carcinoma (DLD-1), α-mangostin induced G1 arrest by downregulating CDK4, CDK2, cyclin D3, and cyclin E, as well as through the upregulation of the p27^kip1^ and p21^waf1/cip1^ CDK inhibitors [40,42]. Besides, in human breast cancer cell line MDA-MB231, G1 arrest induced by α-mangostin was associated with the augmentation of p21^waf1/cip1^, a decreased expression of proliferating cell nuclear antigen (PCNA) and cyclin D1 [38]. On the other hand, and similar to our results, Mizushina et al. [39] reported a cell cycle arrest in the G2/M phase in human colon carcinoma HCT116 cells exposed to 18.5 μM of α-mangostin in 24 h. This arrest was associated with inhibiting cellular DNA topoisomerase I and II activity, suggesting that α-mangostin might be a new type of suppressor of topoisomerase functions. Our data indicated that α-mangostin might affect cell cycle progression, depending on the genetic background.

Although NDGA induced a similar arrest in the G2/M phase in Daoy cells, a transient one in the S phase preceded this arrest. These observations indicate that NDGA could alter the cellular processes involved in at least two different phases of the Daoy cell cycle concomitantly. Previous studies reported that NDGA could arrest cells in different cell cycle phases, but transient arrest in the S phase was not reported previously. For instance, Gao et al. [27] reported that NDGA induced cell cycle arrest at the G1 phase in cervical cancer SiHa cells by upregulating the expression of p21, a key mediator of G1/S transition. Besides, Zavodovskaya et al. [43] found in human breast cancer cells that the growth inhibitory effects of NDGA were associated with an arrest of the cell cycle in the S phase and apoptosis induction. In SH-SY5Y neuroblastoma cells, Meyer et al. [44] reported that NDGA caused cell cycle arrest in sub-G0 in a concentration-dependent manner from 30 to 120 μM after 24 h of treatment. Furthermore, a novel derivative NDGA-based compound, NDGA-P21, could inhibit glioma cell proliferation and arrest the cell cycle in the G1 phase but with poor apoptosis induction [45].

We also documented the proapoptotic properties of α-mangostin and NDGA in Daoy cells through the observed accumulation of the sub-G1 cellular population, a loss of the membrane integrity in flow cytometry assays, and the presence of inter-nucleosomal DNA fragments with TUNEL experiments. Consistent with our results, previous reports showed that α-mangostin and NDGA could induce apoptosis in the diversity of cellular models of cancer. For instance, Li et al. [37] found that α-mangostin at 4 μM induced apoptosis in human breast cancer cells lines (MCF7 and MDA-MB-231), whereas Xu et al. [22] reported the proapoptotic property of α-mangostin at 16 μM in pancreatic cancer cells (BxPC-3 and Panc-1), and Nakagawa et al. [21] reported that α-mangostin induces apoptosis in human colon cancer DLD-1 cells with a concentration of 20 μM. On the other hand, other reports have shown that NDGA and its derivatives could induce apoptosis. For instance, Meyer et al. [46] found a significant increase in PARP cleavage, in a dose-dependent manner, in multiple myeloma cell lines treated with NDGA for 24 h, indicating that NDGA could increase apoptosis. There have also been reports that NDGA in concentrations ranging from 30 μM to 100 μM induced apoptosis in aggressive and metastatic breast cancer cells resistant to HER2 antibody therapy and tamoxifen [26,43]. Further experiments will deeply analyze the mechanism and the pathways involved in these anti-tumoral effects of α-mangostin and NDGA on medulloblastoma cells.

One of the therapeutic approaches against medulloblastoma is chemotherapy such as cisplatin or exisulind, which increases the survival of patients. However, chemotherapy has limitations, such as dosage and efficacy, resistance to therapy, and toxicity side effects. Therefore, it is essential to develop new strategies that reduce these side effects. For example, one strategy would be to use compounds such as α-mangostin or NDGA that, in combination with chemotherapy, induce sensitivity to chemotherapy treatment. Indeed, it has been shown that α-mangostin or NDGA synergizes the effects of cisplatin or exisulind in cervical or lung cancer, respectively [47,48]. Therefore, these compounds α-mangostin 20 μM or NDGA 75 μM could decrease the proliferation of medulloblastoma cells treated with cisplatin or exisulind by increasing the death of cancer cells (compared to cisplatin or exisulind alone). Furthermore, these compounds can be an effective chemosensitizer that selectively damages cancer cells, having less effect on normal cells or healthy tissues.

Taking into account all our data, together with previous reports in the scientific literature that show the ability of both polyphenols to have an effect through the blood–brain barrier, this suggests the potential use of α-mangostin and NDGA as alternative treatments to the conventional therapies against medulloblastoma.

In conclusion, we found evidence that exposure to childhood medulloblastoma-derived cell line Daoy with α-mangostin or NDGA leads to the inhibition of cell growth, characterized by G2/M arrest, in the case of the first compound and a transient arrest in the S phase followed by G2/M arrest for the second one. Both polyphenols induced cell cycle arrest and a clear induction of apoptosis in the Daoy cells. Importantly, α-mangostin exerts more potent suppressive effects in tumoral cell growth and more apoptosis induction than NDGA.

## 4. Materials and Methods

### 4.1. Cell Line and Cell Culture

The Daoy cell line, derived from a primary medulloblastoma tumor, was obtained from the American Type Culture Collection (ATCC number: HTB-186^TM^, Manassas, VA, USA) and maintained in Eagle’s minimum essential medium (EMEM) supplemented with 10% fetal bovine serum (FBS), penicillin–streptomycin solution (100 U.I.–100 μg/mL), and L-glutamine (2 mM) (ATCC, Manassas, VA, USA) at 37 °C in a 5% CO_2_ air humidified atmosphere. NDGA was purchased from Sigma-Aldrich (Toluca, Mexico; catalog number: 74540), whereas *α-mangostin* was obtained as previously described [49]. The purity of *α-mangostin* was 98%, as determined by liquid chromatography-electrospray ionization mass spectrometry (LS-ESI-MS), and the melting point of the compound was 179 to 180 °C, as reported in the literature [50]. NDGA and *α-mangostin* were dissolved in dimethylsulphoxide (DMSO) (ATCC, Manassas, VA, USA) at the final stock concentrations of 100 mM and 20 mM, respectively, and kept at −70 °C and protected from light until used.

### 4.2. Cellular Treatments

For the viability assays, Daoy cells at a density of 1 × 10^5^ cells/mL were seeded in a 6-well plate in 2 mL of culture medium and grown for 24 h. Later, taking as a reference the concentrations of these compounds reported in the literature, the cell cultures were incubated with increasing concentrations of α-mangostin (10, 15, 20, and 40 μM) or NDGA (25, 50, 75, 100, and 200 μM) for 24 h. The concentration range selected for each compound was based on previous reports in the literature. The control cultures were incubated with 0.02% DMSO (*v/v*) for 24 h. In the Cell Cycle and Cell Death analysis case, 1 × 10^6^ cells/mL were grown in 100-mm^3^ culture dishes in a final volume of 10 mL and treated with the indicated concentrations of α-mangostin or NDGA.

### 4.3. Cell Viability Assays

We used crystal violet and trypan blue dyes to determine the effects of α-mangostin and NDGA on the cell viability. In the first case, the culture medium was discharged after cellular incubation with α-mangostin or NDGA, and the attached cells were washed twice with 1 mL of Dulbecco’s phosphate-buffered saline (D-PBS) and were fixed with 1 mL of 1% formaldehyde overnight. Later, the formaldehyde solution was eliminated, and the cells were stained with 1 mL of 0.1% crystal violet for 15 min at room temperature. The plates were rinsed with water to remove the excess dye and air-dried. Next, the dye incorporated into the cells was eluted with 1 mL of 10% acetic acid for 5 min, and a 100-μL aliquoted eluted dye was read on a 96-well plate reader at 570 nm (Multiskan MCC type 355, Fisher Scientific, Vantaa, Finland).

For trypan blue exclusion assays, floating cells were collected from centrifugation and adherent cells were detached with a solution containing 0.25% trypsin–0.53-mM ethylenediaminetetraacetic acid (EDTA) after the indicated treatment with α-mangostin or NDGA. Floating and trypsinized cells were joined and immediately centrifugated at 1500 rpm by 5 min (GPR centrifuge, Beckman Instruments Inc., Palo Alto, CA, USA), washed twice, and resuspended in 1-mL D-PBS. Afterward, 10 μL of cellular suspension was mixed with 10 μL of 0.4% trypan blue dye and counted immediately in a TC20^TM^ automated cell counter (Bio-Rad Laboratories, Inc., Hercules, CA, USA).

### 4.4. Carbonyl Proteins and GSH and GSSG Quantifications

Daoy cells (1 × 10^5^ cells/mL) were seeded in a 6-well plate in 2 mL of culture medium and grown for 24 h. Later, the cells were treated with 20-μM α-mangostin and 75-μM NDGA for 24 h, and both floating and adherent cells were harvested; the cell pellet was kept at −70 °C until it was used. The carbonyl proteins were measured, as previously described by Levine et al. [51]. This method is based on the reaction of oxidized carbonyl protein groups with 2,4-dinitrophenylhydrazine (2,4-DNPH) to produce dinitrophenyl (DNP) hydrazone, which absorbance is measured at λ = 370 nm on an Epoch model multiple plate reader (BioTek Instruments, Winooski, VT, USA). Briefly, the cell extracts of the treatments were incubated for 1h with 10-mM 2,4-DNPH. Then, the proteins were precipitated with 20% trichloroacetic acid. The proteins were washed four times with an ethanol–ethyl acetate mixture (1:1 *v/v*), solubilized in 6-M guanidine hydrochloride, and the absorbance was measured at λ = 370 nm.

The total glutathione (glutathione (GSH) + glutathione disulfide (GSSG)) and GSSG were measured by the method described by Rahman et al. [52]. Briefly, GSH is oxidized by 5,5′-dithiobis-2-nitrobenzoic acid (DTNB) to 5-thio-2-nitrobenzoic acid (TNB; detectable at λ = 412 nm) and TNB glutathione adducts (GS-TNB). Glutathione reductase (GR) reduces GS-TNB and GSSG to GSH, which DTNB oxidizes to TNB. Therefore, the amount of total glutathione calculated signifies the sum of GSH and GSSG. Then, GSSG was also evaluated by the Rahman method mentioned above, where the samples were previously treated with 2-vinylpyridine (2-VP). 2-VP covalently associates with GSH, removing it altogether, leaving GSSG as the only measurable substrate of the assay. Finally, GSH was calculated by subtracting GSSG from the total glutathione (GSH + GSSG). The cell extract of each treatment was diluted with 200 μL of potassium phosphate EDTA (KPE) buffer (0.1-M potassium phosphate and 5-mM disodium EDTA, pH 7.5). Subsequently, two separate samples of 20 μL each and treated with 2-VP were used to measure the total glutathione or GSSG, mixed with DTNB (2.5 mM) and GR (250 U/mL). Finally, β-Nicotinamide adenine dinucleotide phosphate reduced (β-NADPH) was added, and the absorbance at λ = 412 nm was measured at intervals of 60 s for 2 min. The rate of change in absorbance for each experiment was compared with the GSH or GSSG standards.

### 4.5. Cell Cycle Analysis

To study the influence of α-mangostin and NDGA in cell cycle progression, floating and trypsinized cells were harvested after their respective treatment, pelleted by centrifugation, and washed twice with D-PBS. Cellular pellets were resuspended in 1 mL of ice-cold 70% ethanol and maintained at −20 °C overnight. Later, cells were washed twice with D-PBS and incubated with 100 μL of trypsin solution (0.03 mg/mL) for 10 min. Next, the cellular suspension was further incubated with 100 μL of a solution containing 0.5-mg/mL trypsin inhibitor–0.1-mg/mL ribonuclease A for 10 min. Then, the DNA was stained with 80 μL of propidium iodide (0.4 mg/mL) for 30 min. Finally, the cell cycle profile was determined in flow cytometer BD FACSCanto^TM^ II (BD Biosciences, San Jose, CA, USA). Data were analyzed with FlowJo Office software version X (FlowJo, LLC., Ashland, OR, USA). At least 10,000 events were read for each sample.

### 4.6. Cell Death Assay

To evaluate cell death, we used the LIVE/DEAD assay following the indications of the supplier (LIVE/DEAD Fixable Dead Cell Stain Kits, Invitrogen. Eugene, OR, USA). Briefly, after treating the cell cultures as indicated before, floating and adherent cells were harvested and resuspended in 1-mL D-PBS. Then, 1 μL of fluorescent reactive dye was added to the cellular suspension and incubated for 30 min at room temperature. The cells were washed twice with 1 mL of D-PBS with 1% bovine serum albumin and processed in flow cytometer BD FACSAria^TM^ using 488-nm excitation and emission detected with a 610/20 filter (BD Biosciences, San Jose, CA, USA). At least 10,000 events were captured for each sample. An analysis of the results was performed with FlowJo Office software version X and WinList 3D software version 9.0. (Verity Software House, Topsham, ME, USA)

### 4.7. TUNEL Assay

Daoy cells were treated as indicated before to study the proapoptotic potential of α-mangostin and NDGA, and both floating and adherent cells were harvested. The terminal deoxynucleotidyl transferase (TdT) dUTP Nick-End Labeling (TUNEL) assay was performed following the manufacturer’s instructions (APO-BrdU TUNEL assay kit, Molecular Probes, Invitrogen, Eugene, OR, USA). Briefly, cells were resuspended in 5 mL of cold 1% paraformaldehyde in D-PBS and incubated on ice for 15 min. Later, cells were pelleted by centrifugation and washed twice with D-PBS. Cellular pellets were resuspended in 5 mL of ice-cold 70% ethanol and incubated on ice for at least 30 min. Then, the DNA was labeled with bromodeoxyuridine (BrdU) for 60 min and later stained with Alexa Fluor 488-labeled anti-BrdU antibody for 30 min at room temperature. Samples were protected from light during the incubations. The results were analyzed in flow cytometer BD FACSAria^TM^. A minimum of 10,000 events was acquired for each experiment.

### 4.8. Statistical Analysis

All data represented at least three independent experiments and were expressed as the mean ± S.E. and were analyzed using statistical program GraphPad Prism 5 (GraphPad Software Inc., La Jolla, CA, USA). *p*-value < 0.05 was established as significant. Cell viability, cytotoxic effects, and induced apoptosis data were analyzed by one-way analysis of variance and Dunnett’s multiple comparison test. * *p* < 0.0001 vs. control. Cell cycle arrest data were analyzed by two-way ANOVA and Bonferroni posttests. * *p* < 0.05 and ** *p* < 0.001. Carbonyl proteins and the GSH and GSSG data were analyzed by ANOVA and Tukey’s test: * *p* < 0.05, ** *p* < 0.005, and *** *p* < 0.0005.

## Data Availability

Data openly available in a public repository that issues datasets with https://pubmed.ncbi.nlm.nih.gov/, accessed on 24 November 2021.

## Sample Availability

Samples of the compounds α-mangostin and NDGA are available from the authors.
